# Phytochemistry, Bioactivities, Pharmacokinetics and Toxicity Prediction of *Selaginella repanda* with Its Anticancer Potential against Human Lung, Breast and Colorectal Carcinoma Cell Lines

**DOI:** 10.3390/molecules26030768

**Published:** 2021-02-02

**Authors:** Mohd Adnan, Arif Jamal Siddiqui, Walid Sabri Hamadou, Mitesh Patel, Syed Amir Ashraf, Arshad Jamal, Amir Mahgoub Awadelkareem, Manojkumar Sachidanandan, Mejdi Snoussi, Vincenzo De Feo

**Affiliations:** 1 Department of Biology, College of Science, University of Hail, Hail 2440, Saudi Arabia; arifjamal13@gmail.com (A.J.S.); walidsabrimail@gmail.com (W.S.H.); arshadjamalus@yahoo.com (A.J.); 2 Bapalal Vaidya Botanical Research Centre, Department of Biosciences, Veer Narmad South Gujarat University, Surat 395007, India; patelmeet15@gmail.com; 3 Department of Clinical Nutrition, College of Applied Medial Sciences, University of Hail, Hail 2440, Saudi Arabia; amirashrafy2007@gmail.com (S.A.A.); mahgoubamir22@gmail.com (A.M.A.); 4 Department of Oral Radiology, College of Dentistry, University of Hail, Hail 2440, Saudi Arabia; smanojk68@gmail.com; 5 Laboratory of Genetics, Biodiversity and Valorisation of Bioresources, High Institute of Biotechnology, University of Monastir, Monastir 5000, Tunisia; 6 Department of Pharmacy, University of Salerno, Via Giovanni Paolo II, 132, 84084 Salerno, Italy; defeo@unisa.it

**Keywords:** *Selaginella repanda*, breast cancer, lung cancer, colon cancer, phytochemistry, natural product, phytomedicine, pharmacokinetics, ADMET

## Abstract

In this study, we investigated the bioactive potential (antibacterial and antioxidant), anticancer activity and detailed phytochemical analysis of *Selaginella* *repanda (S. repanda)* ethanolic crude extract for the very first time using different in vitro approaches. Furthermore, computer-aided prediction of pharmacokinetic properties and safety profile of the identified phytoconstituents were also employed in order to provide some useful insights for drug discovery. *S. repanda*, which is a rich source of potent natural bioactive compounds, showed promising antibacterial activity against the tested pathogenic bacteria (*S. aureus*, *P. aeruginosa*, *E. coli* and *S. flexneri*). The crude extract displayed favorable antioxidant activity against both 2,2-diphenyl-1-picrylhydrazyl (DPPH) (IC_50_ = 231.6 μg/mL) and H_2_O_2_ (IC_50_ = 288.3 μg/mL) molecules. *S. repanda* also showed favorable and effective anticancer activity against all three malignant cancer cells in a dose/time dependent manner. Higher activity was found against lung (A549) (IC_50_ = 341.1 μg/mL), followed by colon (HCT-116) (IC_50_ = 378.8 μg/mL) and breast (MCF-7) (IC_50_ = 428.3 μg/mL) cancer cells. High resolution-liquid chromatography–mass spectrometry (HR-LC–MS) data of *S. repanda* crude extract revealed the presence of diverse bioactive/chemical components, including fatty acids, alcohol, sugar, flavonoids, alkaloids, terpenoids, coumarins and phenolics, which can be the basis and major cause for its bioactive potential. Therefore, achieved results from this study confirmed the efficacy of *S. repanda* and a prospective source of naturally active biomolecules with antibacterial, antioxidant and anticancer potential. These phytocompounds alone with their favorable pharmacokinetics profile suggests good lead and efficiency of *S. repanda* with no toxicity risks. Finally, further in vivo experimental investigations can be promoted as probable candidates for various therapeutic functions, drug discovery and development.

## 1. Introduction

From decades, plants are well conceded as a source of medicine against different kinds of human ailments [1,2,3]. Several examples of plant-based drugs include nicotine (antimicrobial), nordihydroguaiaretic acid (antioxidant), vinblastine (anticancer), aescin (anti-inflammatory), acetyldigoxin (cardiotonic), l-dopa (antiparkinsonism), etc. In spite of the discovery of innumerable drugs of plant origin, the exploration of novel bioactive compounds is yet requisite to enhance the range access and to search less toxic and more efficacious drugs [4,5,6]. Medicinal and economic importance of higher plants, specifically angiosperms, has been explored thoroughly. However, lycophytes and ferns have been woefully overlooked. Most humanity presumes that there is a little usage of lycophytes and ferns. Though, these plants have delineated plenty of health-related benefits to humanity since ancient times. Their uses are urged in the Ayurvedic (Sushruta, Charka, Samhita), Unani, homeopathic and other systems of medicines. They exert influence on millions of human lives as conventional treatments for various diseases like, burn, cold, ascarid diseases, trauma bleeding, diarrhea and others [7].

The cosmopolitan genus *Selaginella* also acknowledged as a “spike moss” possessing around 700–750 species distributed around the globe. The members of the *Selaginella* are well known for their uses in conventional folk medicine, food, handicrafts and as ornaments. The *Selaginella* plants are usually used by the tribal community to cure fever, jaundice, hepatic disorders, cirrhosis, diarrhea, cholecystitis, sore throat, cough of lungs, promote blood circulation, remove blood stasis and stops external bleeding after trauma and after separation of the umbilical cord [8]. Few species of *Selaginella* such as, *S. tamariscina*, *S. lepidophylla*, *S. chrysocaulos*, *S. bryopteris*, *S. labordei* and *S. moellendorffii* have been reported for their in vitro antimicrobial, antiviral, antidiabetic, antimutagenic, anti-inflammatory, antinociceptive, antispasmodic and anticancer potentials due to the high content of different phytochemicals, such as flavonoids, phenylpropanoids, steroids, pigments, oxygen heterocycle, lignans, coumarins, quinoids, chromones, benzenoids, carbohydrates and alkaloids [9,10,11,12,13,14,15,16,17,18]. However, very few information related to *S. repanda* phytochemistry is available, though it has been reported as an important ethnomedicinal fern.

Therefore, this study aims to evaluate the bioactivities (antibacterial, antioxidant and anticancer activity against human lung, breast and colon cancer cell lines) of *S. repanda* for the first time. In order to evaluate the potency and bioactive potential, *S. repanda* crude extract was also analyzed by high resolution-liquid chromatography–mass spectrometry (HR-LC–MS) in detail to identify the phytochemical constituent’s being the reason for its bioactivity. Obtained results will support the further in vivo studies for the possible therapeutic usage of *S. repanda* against various diseases/ailments and in the treatment of metastatic cancer.

## 2. Results and Discussion

Medicinal plants have been utilized chiefly as ethnic remedies against various diseases since ancient time. They are extensively contemplated as a source of novel phytochemicals with bioactive potential. In order to treat a variety of diseases, these phytomedicines can also lead in drug discoveries or itself become a potential drug. Hence, it is very essential to determine and estimate the bioactive potential of ethnomedicinal plants by particularizing the phytochemistry and developing them as a potent source for therapeutic agents [19,20,21]. Pteridophytes ethnomedicinal nature makes them as a traditional medicine to cure several complicated health conditions [22,23,24]. Therefore, in the presented work, we evaluated the antibacterial, antioxidant and anticancer properties of *S. repanda*.

### 2.1. Antibacterial Potential of S. repanda

In recent times, almost all developing and developed countries are going through with a global problem of antibiotic resistance, which is continuously challenging the global healthcare sector. Multidrug-resistant pathogens are emerging and spreading in such a way that it continues to diminish the current antimicrobial therapies. This demands a search for novel antimicrobial substances, preferably from natural sources. Plants are one such example consisting of a variety of bioactive compounds with known therapeutic properties [3]. The microdilution method was used to study the antagonistic potential of *S. repanda* crude extract against pathogenic bacterial strains *S. flexneri, S. aureus, E. coli* and *P. aeruginosa*. Results revealed that *S. repanda* showed significant antagonistic activity against all the four tested bacterial strains, and are presented in Table 1.

The antimicrobial activity of different species of *Selaginella* was evaluated against various human pathogenic bacteria. The crude extract of *S. bryopteris* was reported for its antibacterial activity against different Gram-negative and Gram-positive bacteria such as *E. coli*, *E. faecalis*, *C. tropicalis*, *S. aureus*, *C. albicans*, *C. krusei* and *K. pneumonia* [25]. *S. equalifolia* and *S. involvens* has been reported to show antimicrobial activity against different poultry pathogens namely, *Klebsiella*, *Salmonella*, *Staphylococcus*, *Proteus* and *Bacillus* [26]. *S. inaequalifolia* showed good antagonistic activity against *S. aureus*, *E. coli* and *C. albicans* [27]. *S. convoluta* showed significant antibacterial potential against *B. cereus*, *E. coli*, *S. enterica*, *S. marcescens*, *K. pneumoniae*, *S. flexneri*, *E. faecalis* and *S. aureus* [28]. *S. tamariscina* is known to possess potent activity against oral bacterial pathogens such as *P. gingivalis*, *P. intermedia*, *S. mutans*, *S. sobrinus*, *S. gordonii*, *F. nucleatum*, *S. sanguinis*, *S. anginosus*, *S. ratti*, *A. actinomycetemcomitans*, *S. parasanguinis*, *S. criceti* and *S. downei* [29]. Our study is also in line with previous antibacterial studies and showed that *S. repanda* possess antibacterial potential against various human pathogenic bacteria.

### 2.2. Antioxidant Potential of S. repanda

Free radicals are naturally formed in our body and perform many cellular processes [30]. At a higher concentration, free radicals can be dangerous and can damage the important constituents of the cell, which may include cell membrane, proteins, DNA, etc. Damage to DNA, in particular, may lead to cancer or other severe health conditions. However, in order to prevent this type of cellular damage, antioxidants play an essential role. They are generally described by “mopping up” free radicals, which means they are able to neutralize the electrical charge and can prevent the free radical from receiving electrons from other molecules [30]. It has been reported that plant extract or naturally extracted compounds having antioxidant activity, exhibits anticancer activity in a defined mechanism.

**Figure 1:**
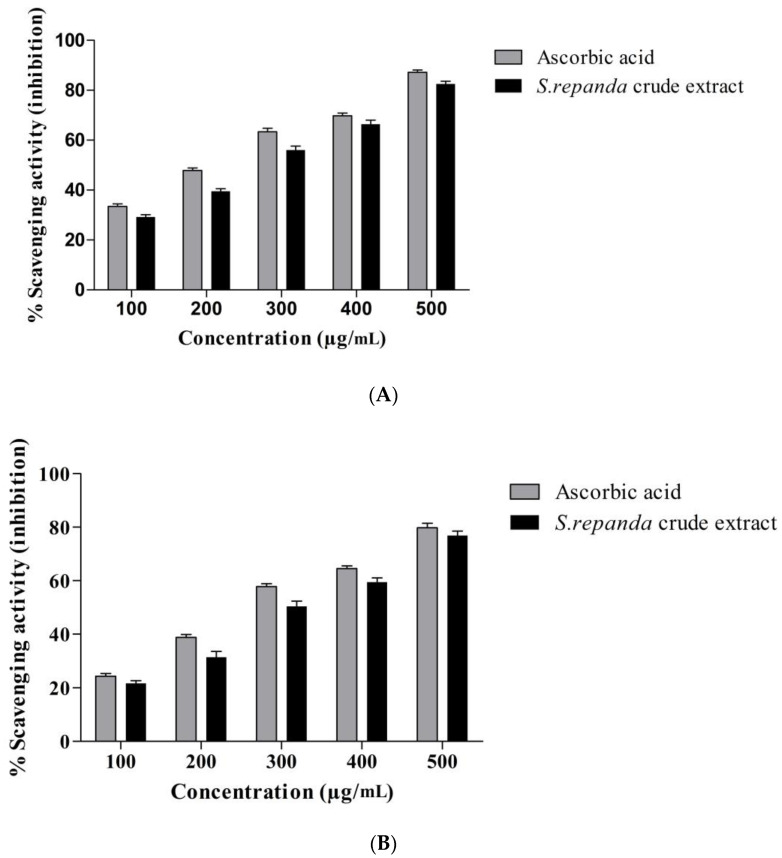
Antioxidant activity of ascorbic acid (standard) and *S. repanda* crude extract against (**A**). DPPH molecules. (**B**). H_2_O_2_ molecules. Error bars indicate SDs (±standard deviation) of three independent experiments (*p* < 0.05).

*S. repanda* antioxidant potential was analyzed against DPPH and H_2_O_2_ molecules in comparison to ascorbic acid. The results of antioxidant activity displayed that *S. repanda* crude extract exhibited remarkable free radical scavenging activity, both against DPPH and H_2_O_2_ molecules in a dose dependent manner. The scavenging capacity against DPPH free radicals was higher than H_2_O_2_ molecules. The IC_50_ values of crude extract of *S. repanda* for DPPH and H_2_O_2_ were 231.6 μg/mL and 288.3 μg/mL, respectively (Figure 1A,B). The antioxidant potential of different species of *Selaginella* have been evaluated by different methods; from which, most are based on the determination of free radical scavenging activity. Commonly used methods are, ABTS, DPPH, superoxide anion radical scavenging assays and total phenolic content. Total phenolic content of *S. repanda* dried powder was found to be 6.51 ± 0.71 mg gallic acid equivalents/g dry matter. Indian Sanjeevani (*S. bryopteris*) is well known for its protective effect against various stress-induced conditions [31]. The crude extract of *S. tamariscina* have strong antioxidant property, as its extract can reduce blood sugar levels and also able to act as a lipid peroxide and increases insulin serum [32]. In one study, the aqueous extract of *S. involvens*, *S. delicatula* and *S. wightii* also displayed in vitro lipid peroxidation and varying levels of hydroxyl radical scavenging activity. The 50% inhibition (EC_50_) for in vitro lipid peroxidation of *S. wightii, S. delicatula* and *S. involvens* was 76.6 ± 4, 38.2 ± 1.2 and 2.1 ± 0.1, respectively. Moreover, flavonoids obtained from the *S. doederleinii* also possess very strong free radical scavenging activity [33]. Similar results were found in our study. *S. repanda* displayed potent antioxidant activity, indicating its protective role against free radicals.

### 2.3. Anticancer Potential of S. repanda

Cancer is the main health burden in developing and developed countries, as it is the second leading cause of deaths globally. Different types of treatments are available for the therapy, depending on the stage of cancer and type. Radiotherapy and surgery are efficacious in the treatment of cancer at the earlier stage, while chemotherapy was used when the tumor reaches the stage of metastasis. However, the high cost of treatments is associated with the side effects, which accounted for the use of herbal products for the treatment of different types of cancers. Over the past decade, natural products perceived high attention because of their capability as novel therapeutic and preventive agents. About 60% of all orthodox anticancer drugs are directly or indirectly derived from plants [34], which imply that medicinal plants have the ability in leading to the discovery of novel drugs.

**Figure 2:**
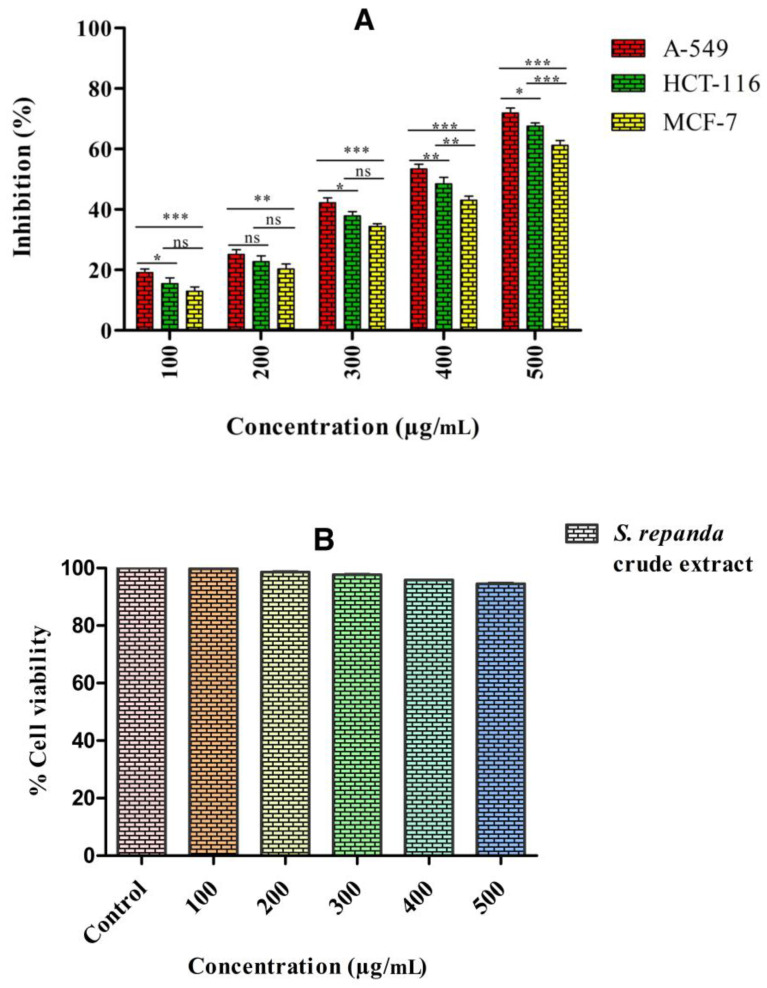
(**A**) Anticancer activity against three different human cancer cell lines, i.e., breast (MCF-7), colon (HCT-116) and lung (A549). (**B**) Cytotoxicity of *S. repanda* crude extract against human normal colon cells (CRL-1831). Error bars indicate SDs (± standard deviation) of three independent experiments. NS > 0.05, * *p* < 0.05, ** *p* < 0.005, *** *p* < 0.0005

*Selaginella* species have different groups of chemical compounds, which are well known to have a wide-range of biological actions and are a potential source for finding the novel anticancer drugs. As many of the phytochemical compounds obtained from few of these species possess strong cytotoxic activity against various cancer cell lines. *S. repanda* crude extract was evaluated for its anticancer potential by MTT assay against three different human cancer cell lines, i.e., breast (MCF-7), colon (HCT-116) and lung (A549). Our results revealed that, proliferation of all the three cell lines were inhibited by *S. repanda* in a dose dependent manner, compared to fluorouracil, which was used as the positive control. The crude extract of *S. repanda* showed the highest growth inhibitory activity against A549 cells with IC_50_ value of 341.1 μg/mL, followed by HCT-116 cells (378.8 μg/mL) and MCF-7 cells (428.3 μg/mL) (Figure 2A). Cytotoxicity test of *S. repanda* crude extract against non-cancerous human normal colon cells (CRL-1831) was also performed and results revealed no cytotoxicity to normal cells (Figure 2B).

One study revealed the anticancer effect of ethanolic and aqueous extracts of *S. doederleinii* using the brine shrimp lethality test against two cancer cell lines MDAMB231 (breast) and HepG2 (liver). As a result, after 24 h of exposure to 50% lethal concentration (LC_50_) in the brine shrimp lethality test was found to be >1000 μg/mL. MDAMB231 and HepG2 were found to be the most susceptible with the treatments of ethanol (LC_50_ = 306 μg/mL) and aqueous (LC_50_ = 329 μg/mL) extracts of *S. doederleinii*, respectively [35]. The crude extract of *S. tamariscina* also showed potent anticancer activity against different cancer cell lines. It was found to decrease the metastasis, expression of MMP-2 and 9 (matrix metalloproteinase) and urokinase plasminogen activator in A549 cells and Lewis lung carcinoma [36]; inhibits nucleus antigen cell from stomach epithelium [37]; inhibits gastric cancer cells [37] and induce apoptosis via blockade of fatty acid synthesis in breast cancer [38]; inhibits leukemia cancer cells HL-60 and U937 [39];. Apart from this, amentoflavone was extracted from the crude extract of *S. tamariscina* and its anticancer efficacy was screened against five different cancer cells, including HL-60 (human leukemia cells), HeLa (human cervical carcinoma cells), PANC-1 (human pancreatic cancer cells), MCF-7 (human breast cancer cells) and BEL-7402 (human hepatoma carcinoma cells). The extract was found to be efficient in the inhibition of the proliferation of all cells with a remarkable inhibition of HL-60 [39].

The cytotoxic effect and apoptosis induction potential of hexane, methylene chloride, ethyl acetate and butanol extracts of *S. plana* was performed against MCF-7 cells. Different crude extracts of *S. plana* displayed inhibition of MCF-7 cells with an IC_50_ value of 30 μg/mL, 19 μg/mL, 24 μg/mL and 2 μg/mL respectively. Butanol crude extract was found as the highest cytotoxic and apoptotic induction against MCF-7 cancer cells [40]. The cytotoxic and apoptosis activity of three different crude extracts (ethyl acetate, ethanol and aqueous) of *S. uncinata*, *S. tamariscina*, *S. remotifolia*, *S. delicatula*, *S. moellendorfii*, *S. pulvinata* and *S. labordei* were evaluated using BEL-7402, HT-29 and HeLa cells. In results, *S. labordei*, *S. tamariscina* and *S. uncinata* had a higher inhibition of Bel-7402 and HeLa cells, whereas, *S. moellendorfii* had moderate inhibition, but *S. remotifolia* and *S. pulvinata* had almost no inhibitory activities. The major bioactive compounds responsible for the inhibition for cancer cells were bioflavonoids, detected in the ethyl acetate extracts. Moreover, the efficacy of all three extracts of all the plants on cell inhibition and apoptosis were not the same, they were highly efficient on HeLa cells than HT-29 cells [41]. Our results also revealed similar data, *S. repanda* possessing anticancer potential against three malignant lineages, HCT-116, MCF-7 and A549 cell lines. Though, all the three cell lines have high metastatic potential, *S. repanda* was found to inhibit the proliferation of all three malignant cells in a time and concentration dependent manner.

### 2.4. Identification of Phytochemical Compounds of S. repanda Using HR-LC–MS

**Figure 3:**
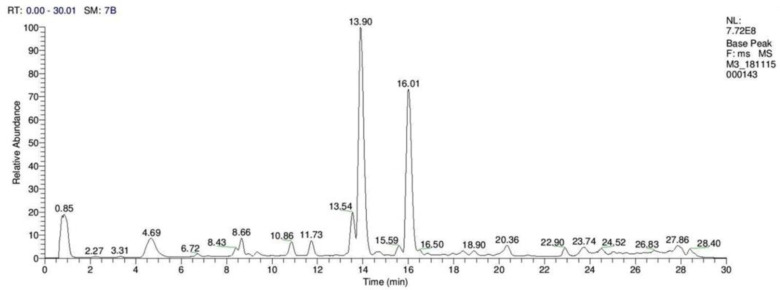
High resolution-liquid chromatography–mass spectrometry (HR-LC–MS) spectrum of *S. repanda* crude extract.

**Figure 4:**
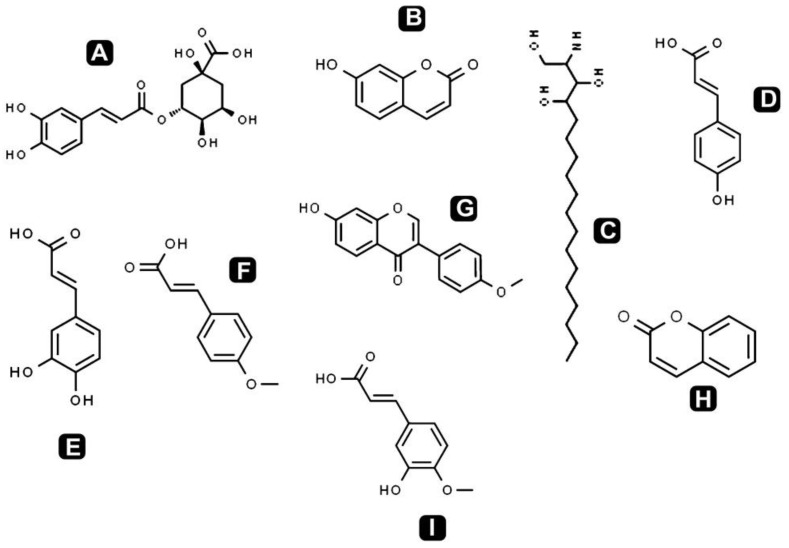
Chemical structures of the identified phenol compounds in *S. repanda* crude extract. **A.** Chlorogenic acid **B.** 7-Hydroxycoumarine **C.** 2-amino-1,3,4-octadecanetriol **D.** 4-coumaric acid **E.** Caffeic acid **F.** 4-Methoxycinnamic acid **G.** Formononetin **H.** Coumarin **I.** Isoferulic acid.

**Figure 5:**
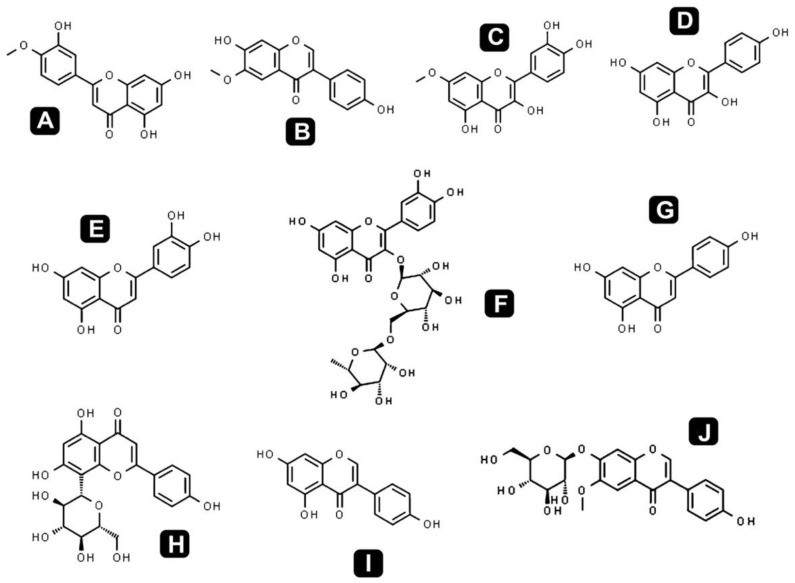
Chemical structures of the identified flavonoid compounds in *S. repanda* crude extract. **A.** Diosmetin **B.** Glycitein **C.** Rhamnetin **D.** Kaempferol **E.** Luteolin **F.** Rutin **G.** Apigenin **H.** Vitexin **I.** Genistein **J.** Glycitin.

**Figure 6:**
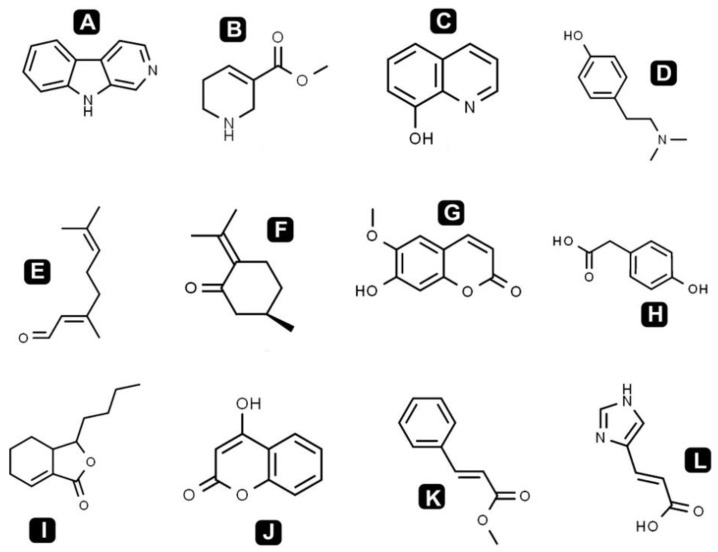
Chemical structures of the identified alkaloids, terpenoids, coumarins, benzenoids, esters and monocarboxylic acid compounds in *S. repanda* crude extract. **A.** Norharman **B.** Guvacoline **C.** 8-Hydroxyquinoline **D.** Hordenine **E.** Citral **F.** Pulegone **G.** Scopoletin **H.** 4-Hydroxyphenylacetic acid **I.** Sedanolide **J.** 4-Hydroxycoumarine **K.** Methylcinnamate **L.** Urocanic acid.

Different classes of phytochemicals are omnipresent in plants and have been linked with diverse biological activities. This study revealed the presence of diverse groups of chemical compounds, which are known to possess a broad range of bioactivities. After the evaluation of antibacterial, antioxidant and anticancer activities, a comprehensive phytochemical analysis from crude extract of *S. repanda* was carried out via UHPLC-PDA-ESI–MS/MS. Chromatogram was obtained with both positive and negative run and different types of phytochemicals were identified (Figure 3). Different classes of metabolites such as sugars, amino acids, vitamins, alkaloids, flavonoids, terpenoids, phenols, etc., were detected (Figure 4, Figure 5 and Figure 6).

Flavonoids and natural phenolic acids are one of the most prevalent and pharmacologically active groups of plant secondary metabolites. In this study, *S. repanda* crude extract revealed the presence of phenols like coumarin, chlorogenic acid, 7-hydroxycoumarine, caffeic acid, 4-methoxycinnamic acid, isoferulic acid, formononetin, 2-amino-1,3,4-octadecanetriol and 4-coumaric acid. The other main flavonoids identified in the present study were rutin, vitexin, quercetin, quercetin-3β-d-glucoside, kaempferol, apigenin, luteolin, rhamnetin, glycitein, diosmetin and genistein. They are well known for their potential therapeutic applications including antioxidant, anti-inflammatory, anticancer, cardioprotective effects, etc. These compounds play a crucial role in prevention of cancer through various mechanisms at the molecular level. This may include impeding the signaling pathways, migration, differentiation and proliferation inhibition, gene regulation, carcinogen metabolism, induction of apoptosis via arresting cell cycle, etc. [42].

Alkaloids are nitrogenous compounds, which are widely distributed from prokaryotes to eukaryotes and are well-known for their different biological activities like anti-microbial, anti-HIV, anticancer and antiparasitic [43]. In the present study, 8-hydroxyquinoline, norharman, hordenine and guvacoline were identified. Two terpenoids, citral and pulegone were also identified from the crude extract of *S. repanda*. Terpenoids possess diverse biological activities including antimalarial, antioxidant, antibacterial, antiviral, anticancer and anti-inflammatory. They are also used for the treatment of various diseases [20]. Apart from this, coumarins, different amino acids, fatty acids, sugars and sugar alcohol were also detected. Detailed list of identified compounds with their mass (*m*/*z*), retention time and bioactivities are presented in Table 2.

Therefore, our data suggested that the presence of these diverse biomolecules in *S. repanda* crude extract is the major reason for its antibacterial, antioxidant and anticancer activities. Our results provide an opportunity to explore further this medicinally treasured pteridophyte. Moreover, for the first time we studied that the *S. repanda* crude extract had a strong dose dependent cytotoxicity against three malignant cancer cell lines (HCT-116, MCF-7 and A549). Further studies are needed in order to demonstrate the efficacy and potency of specific compound in the crude extract, which might be responsible for the particular biological activity.

### 2.5. Pharmacokinetic and Toxicity (ADMET) Profiles of Identified Phytoconstituents from S. repanda Ethanolic Crude Extract

It is well known that good drug activity may be destroyed due to inadequate ADMET (absorption, distribution, metabolism, excretion and toxicity) properties. In addition, unwanted pharmacokinetics and toxicity are important reasons for the failure of drug discovery in the clinical phase, which is very costly. In order to decide, the possibility of *S. repanda* ethanolic crude extract to become or not become a good candidate for suitable drug, ADMET parameters have been assessed using in silico tools (Table 3). Interestingly, all identified phytoconstituents were found to meet the Lipinski’s rule of five, and some of them such as hordenine, 4-coumaric acid and Diosmetin follows also Ghose, Veber and Egan filters with most of them displaying a good bioavailability score (55–85%). The solubility as an important feature for the absorption of the molecule and its distribution in the body given by the values of aqueous solubility indicates most of the compounds were highly soluble in water and the others are soluble.

To be a transdermal drug delivery, the skin permeability, which defines the rate of a chemical penetrating across the stratum corneum, will be checked to improve its efficacy. A molecule will perfectly penetrate the skin if log Kp was higher than −2.5 cm/h. All phytocompounds seem to possess moderate to good skin penetrability among them, those of scopoletin, sedanolide, (-)-Caryophyllene oxide and hordenine. The Caco-2 cell line is composed of human epithelial colorectal adenocarcinoma cells. Caco-2 permeability can predict the intake of oral drugs as Caco-2. All compounds displayed moderate to potent (log Papp values >0.90 cm/s) Caco-2 permeability values. Among them, scopoletin, sedanolide, (-)-Caryophyllene oxide and hordenine were predicted to have the strongest Caco-2 permeability.

P-gp is a key to estimate active efflux through biological membranes and known as the most important member among ATP-binding cassette transporters or ABC-transporters used to protect the central nervous system (CNS) from xenobiotics. None of the selected phytocompounds were P-gp inhibitor/substrate. Intestinal absorption was also assessed and most of the identified compounds were highly absorbed by the intestine. The skin permeation as given by LogKP was predicted and results demonstrated that identified compounds might be moderately or highly promoted to penetrate through the skin, which confirmed their drug-like properties. Many of them such as Sedanolide, and (-)-Caryophyllene oxide with log BB (logarithm value of brain to plasma concentration ratio) greater than 0.3 were subjected to have more potential to cross brain blood barrier (BBB). Only fewer compounds like arachidonic acid, hexadecanamide and hordenine have the ability to penetrate to CNS. (-)-Caryophyllene oxide and hordenine are amongst the phytoconstituents that will be more distributed with distribution volume logVDss of 0.564 L/kg and 0.887 L/kg in the tissues, respectively.

Human cytochrome P450 (CYP) isoforms involved in drug metabolism in liver were also generated. Amongst them, CYP3A4 is the major and most clinically relevant drug-metabolizing enzyme in the human body. Its inhibition could lead to drug toxicity, drug–drug interactions and other adverse effects. Some of them were non-inhibitors/substrate of any isoenzymes like sedanolide, however as a good result, more than 92% were found to be non-inhibitors of CYP3A4, isoenzyme responsible for the metabolism of about 60% of xenobiotics including drugs, carcinogens, steroids and eicosanoids.

To determine the excretion routes, the total clearance (CLTOT) for both hepatic and renal and renal organic cation transporter 2 (OCT2) substrate as expressed in log ml/min/kg were predicted. Results showed that about 98% of the identified phytocompounds exhibited a positive total clearance values and can be easily excreted.

The toxicity profile of all the identified phytoconstituents from *S. repanda* ethanolic crude extract has been predicted based on AMES toxicity, hepatotoxicity, hERG potassium channel inhibition and skin sensitization parameters. Results outlined that, out of the fifty four identified compounds, only three have a deviated mutagenic and hepatic toxicity potential, which means that about 95% are devoid of any risk of toxicity, 100% have no hERG I inhibition and 75% exerted no skin-sensitive effects.

**Figure 7:**
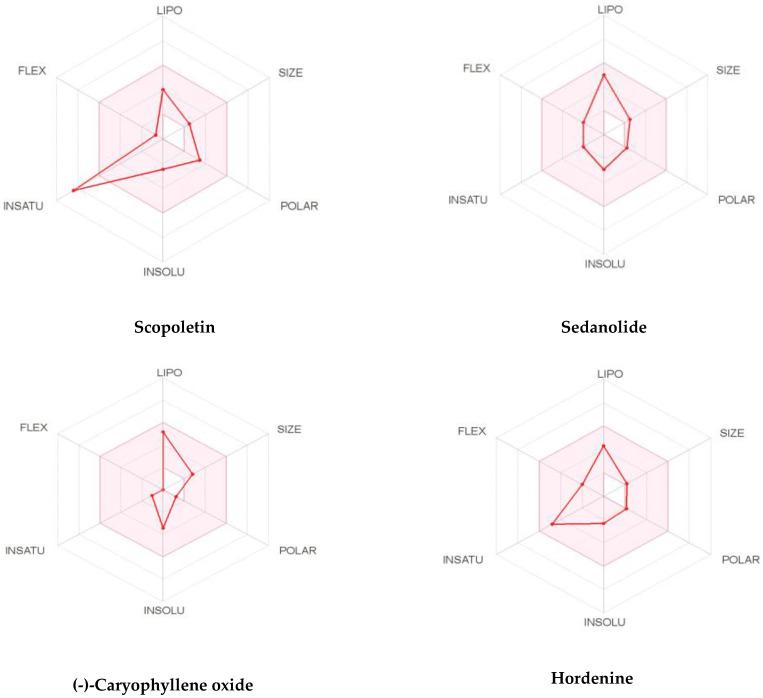
Top bioavailability radar of the phytocompounds based on physicochemical indices ideal for oral bioavailability.

To achieve more information for better bioavailability and drug-likeness of the identified compounds, we present an example of the top four suitable phytocompounds that can be delivered more as a potent candidate for drug discovery (Figure 7). The results of the bioavailability radar have been depicted by the lipophilicity: XLOGP3 between −0.7 and +5.0, size: MW between 150 and 500 g/mol, polarity: TPSA between 20 and 130 Å2, solubility: log S not higher than 6, saturation: fraction of carbons in the sp3 hybridization not less than 0.25 and flexibility: no more than 9 rotatable bonds with the colored zone defined the desired physicochemical space for good oral bioavailability indicating that they possess good drug-likeness properties.

**Figure 8:**
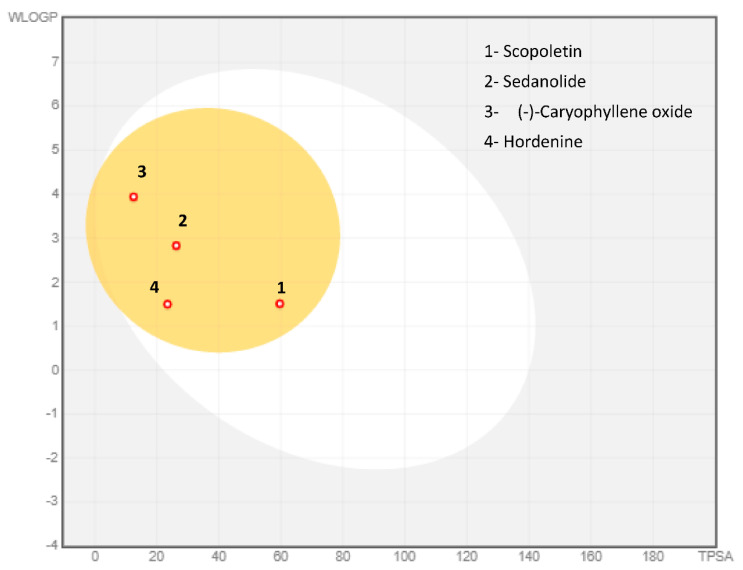
BOILED-Egg model of the volatile constituents using the Swiss ADME predictor.

An example of the BOILED-Egg model (Brain or IntestinaL EstimateD permeation method) (Figure 8) prediction of gastrointestinal (GI) absorption and BBB permeation of the same selected compounds have been exploited. Results shows that the compounds appeared with a red point in the yellow ellipse having high probability of brain penetration and are non-substrate of P-gp (PGP-).

## 3. Materials and Methods

### 3.1. Plant Collection, Storage and Sequence Deposition

**Figure 9:**
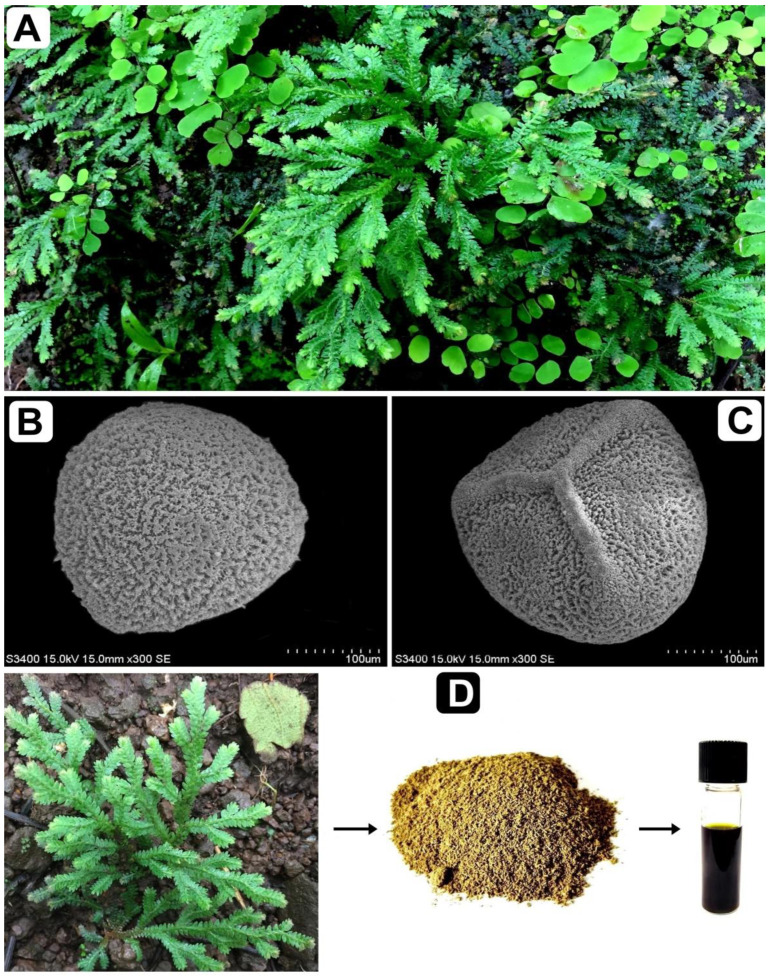
*S. repanda* plant: (**A**) in the wild, (**B**,**C**) the structure of spores under scanning electron microscope (SEM) and (**D**) preparation of the crude extract.

*S. repanda* whole plant was collected from the wild regions of Gujarat state, India during the period of July–August 2020. The plant was identified by the molecular sequencing method, plus by its taxonomic characters (Figure 9). The voucher specimen (BVBRC035) was deposited at bapalal vaidya botanical garden, department of biosciences, Veer Narmad South Gujarat University, Surat, Gujarat, India. *rbcL* gene nucleotide sequence (accession number MT795925) was deposited to NCBI. The whole plant was dried in an oven, followed by grinding into a fine powder and was then stored airtight containers. The percentage yield of the plant extract was calculated according to the following formula:

(1) $$\mathrm{Percentage}\;\mathrm{yield}\;\mathrm{of}\;\mathrm{the}\;\mathrm{extract}=\frac{\mathrm{Weight}\;\mathrm{of}\;\mathrm{extract}\;\left(g\right)}{\mathrm{Weight}\;\mathrm{of}\;\mathrm{plant}\;\mathrm{material}\;\left(g\right)}\times 100$$

### 3.2. S. repanda Crude Extraction

Ethanol (85%) was used for soaking of 20 g *S. repanda* powder for 24 h at 37 °C with vigorous shaking. Whatman no. 1 filter paper was used for filtering the ethanol phase mixture and then concentrated using a rotary evaporator to get the dried residue. Stock solution of crude ethanolic extract was then further used for performing various biological activities such as antibacterial, antioxidant and anticancer.

### 3.3. Antibacterial Assay

#### 3.3.1. Bacterial Strains

Common pathogenic bacterial strains; *S. flexneri* (MTCC 1457), *S. aureus* (MTCC 96), *E. coli* (MTCC 9537) and *P. aeruginosa* (MTCC741) was used to carried out the antibacterial activity. They were obtained from the Microbial Type Culture Collection (MTCC), Chandigarh, India, and maintained on Muller–Hinton agar (MHA). To bring up to 0.5 McFarland standard 10^8^ colony forming units/mL (CFU/mL), sterile saline solution was used and culture turbidity was adjusted.

#### 3.3.2. Microdilution Method

Antibacterial activity of *S. repanda* crude extract was performed in 96-well microtiter plates against pathogenic bacteria, as described previously [83]. The inoculums were prepared from a 6 h Muller–Hinton broth (MHB) culture, and suspensions were adjusted to 0.5 McFarland turbidity standards (10^8^ CFU/mL). *S. repanda* crude extract was diluted to twofold ranging from 1000 to 0.48 µg/mL (80 µL as final volume) with a DMSO concentration >1%. Afterward, 20 µL of bacterial suspensions and 100 µL of MHB were loaded onto microtiter plates. Plates were then incubated at 37 °C for 24 h. At the end of the incubation period, microtiter plates were read using a spectrophotometer at 620 nm. Chloramphenicol, a standard antibiotic, was used as a positive control. MHB + DMSO was used as a vehicle control, and MHB alone was used as a sterility control. MIC was recorded as the plant extract with the lowest concentration and has shown absolute inhibition of observable growth [84].

### 3.4. Antioxidant Assays

#### 3.4.1. Determination of Free Radical Scavenging Effects of Antioxidants Using the DPPH Method

*S. repanda* crude extract antioxidant activity was measured against DPPH free radicals in relation to radical scavenging capability [85]. Crude extract of varied concentrations (100–500 μg/mL) were mixed in the tubes containing 2 mL of DPPH solution (6 × 10^−5^ M) in dimethyl sulfoxide (DMSO), followed by dark incubation for 1 h. After incubation, a decrease in absorbance was measured at 517 nm. As a standard, ascorbic acid was used; as a blank, DMSO was used; and as a control, DPPH solution without crude extract was used. Calculation of the percentage of scavenging of DPPH free radicals was then estimated as follows:

DPPH scavenging activity (%) = (A_0_ − A_1_)/A_0_ × 100 (2)

whereas,

A_0_ = absorbance of the control (3a)

A_1_ = absorbance of the sample (3b)

#### 3.4.2. Hydrogen Peroxide (H_2_O_2_) Scavenging Assay

H_2_O_2_ scavenging activity was measured as per the method used by Adnan et al. (2018) [19]. One milliliter of crude extract (100–500 μg/mL) was mixed with 1 mL of 2 mM H_2_O_2_ solution, prepared in 0.1 M phosphate buffer (pH 7.4). The tubes were then incubated for 10 min at room temperature, and absorbance was measured at 230 nm. Absorbance was determined against a blank solution (phosphate buffer without H_2_O_2_). Standard ascorbic acid was used as positive control. Calculation of the percentage of scavenging of H_2_O_2_ was then estimated as follows:

Inhibition (%) = (A_0_ − A_1_)/A_0_) × 100 (4)

whereas,

A_0_ = absorbance of the control (5a)

A_1_ = absorbance of the extract/standard (5b)

### 3.5. Total Phenolic Content of S. repanda

The concentration of total phenolics in the extract was analyzed by the Folin–Ciocalteu colorimetric assay [86]. Firstly, crude extract (0.2 mL), deionized water (0.8 mL) and Folin–Ciocalteu reagent (0.1 mL) was incubated at room temperature for 3 min and then 0.3 mL of Na_2_CO_3_ (20% *w/v*) was added and the mixture was incubated at room temperature for 2 h. Absorbance of the mixture was read at 765 nm. A standard curve of gallic acid from 0 to 100 mg/L was prepared. Total phenolic content was expressed in mg gallic acid equivalents/g dry matter.

### 3.6. Cytotoxicity and Anticancer Assay (MTT Assay)

Cytotoxicity of *S. repanda* crude extract was performed against human normal colon cells (CRL-1831) and anticancer potential was performed against human lung (A549), breast (MCF-7) and colon (HCT-116) cancer cell lines. Cells were obtained from the NCCS, India. They were propagated in a humidified (5% CO_2_) atmosphere at 37 °C, and were maintained in 25 cm^2^ flask containing DMEM supplemented with 10% FBS. They were grown up to 80% confluence and were seeded in 96-well plates at a density of more than 1 × 10^5^ cells per well with incubation conditions mentioned above. Cells were then stained with 0.4% Trypan Blue and viable cell numbers were calculated using a hemocytometer. However, assay was performed as triplicate wells for each concentration. Different concentration of *S. repanda* crude extract (100–500 μg/mL) was then used to treat the cells for 24 h. Followed by washing with PBS solution and subjected with 100 μL of MTT solution (3-(4,5-dimethylthiazolyl-2)-2,5 diphenyltetrazoliumbromide) (5 mg/mL), followed by incubation for 4 h. Finally, the medium was removed and 100 μL of DMSO was added to solubilize the formazan crystals. ELISA reader was then used to determine the amount of formazan crystal by measuring the absorbance at 570 nm. Fluorouracil (5FU) was used as a positive control. All assays were done in triplicate and 50% cytotoxic concentration (IC_50_) was calculated [5].

### 3.7. HR-LC–MS Analysis

Phytochemistry of *S. repanda* crude extract was analyzed using UHPLC-PDA-Detector Mass Spectrophotometer (HR-L=CMS 1290 Infinity UHPLC System), Agilent Technologies^®^, Santa Clara, CA, USA. The liquid chromatographic system consisted of the HiP sampler, binary gradient solvent pump, column compartment and quadrupole time of flight mass spectrometer (MS Q-TOF) with the dual Agilent Jet Stream Electrospray (AJS ES) ion source. Of the sample 10 µL was injected into the system, followed by separation in the SB-C18 column (2.1 mm × 50 mm, 1.8 µm particle size). Solvent A (1% formic acid in deionized water) and solvent B (acetonitrile) were used as solvents. Flow rate of 0.350 mL/min was used, while, MS detection was performed in MS Q-TOF. Compounds were identified via their mass spectra and their unique mass fragmentation patterns. Compound Discoverer 2.1, ChemSpider and PubChem were used as the main tools for the identification of the phytochemical constituents [5].

### 3.8. ADMET Analysis

Prediction of the pharmacokinetics and toxicity of the identified compounds from the *S. repanda* ethanolic crude extract was performed using the SwissADME (http://www.swissadme.ch/) and pkCSM (http://biosig.unimelb.edu.au/pkcsm/prediction) online tools [87,88,89].

### 3.9. Statistical Analysis

Results are expressed as mean ± SD of the number of experiments performed. A Student’s *t*-test for paired or unpaired values was performed and a *p*-value of <0.05 was considered statistically significant. Statistical analysis was conducted with software GraphPad Prism Version 7.03.

## 4. Conclusions

From this current work, it was concluded that *S. repanda* possessed strong antibacterial activity against different pathogenic bacterial strains of human interest, promised a source of antioxidant compounds and offered a worthy understanding about its anticancer potential. Moreover, *S. repanda* can also be used as a possible ingredient, nutraceutical or functional food, which requires further exploration, in vivo pharmacological and toxicological studies, to prove the unexplored beneficial aspects of *S. repanda* significant medicinal properties. In silico results indicated a good pharmacokinetic and safety profile of the phytochemical constituents, which make *S. repanda* ethanolic crude extract a potential drug candidate for the treatment of many diseases.

## Figures and Tables

**Table 1:** Antibacterial activity of *S. repanda* crude extract.

Bacterial Strain	*S. repanda* Crude Extract (µg/mL) (MIC)	Chloramphenicol (µg/mL) (MIC)	Negative Control (85% Ethanol)
*E. coli*	31.25	7.812	-
*S. aureus*	62.5	15.625	-
*P. aeruginosa*	125	31.25	-
*S. flexneri*	250	62.5	-

**Table 2:** Profiling of bioactive compounds present in *S. repanda* crude extract with their biological activity.

Compound	Formula	Class of Phytochemical	*m/z*	RT (min)	Mass	Mode of Action	References
Choline	C_5_H_13_N O	essential nutrient (vitamin)	111.4	0.798	103.09988	-	-
α-Lactose	C_12_H_22_O_11_	sugar	349.9	0.839	342.11521	-	-
Acetylcholine	C_7_H_15_ NO_2_	essential nutrient (vitamin)	149.7	0.850	145.11	-	-
Betaine	C_5_H_11_NO_2_	amino acid	122.2	0.935	117.07901	Antimicrobial, antioxidant, antiangiogenesis	[44]
l(-)-Carnitine	C_7_H_15_NO_3_	amino acid derivative	157.6	0.93	161.10489	*-*	-
d-Glucosamine	C_6_ H_13_ NO_5_	amino sugar	183.3	0.946	179.079	Antitumor	[45]
l-Pyroglutamic acid	C_5_H_7_NO_3_	amino acid	133.1	1.04	129.0425	-	-
l-Norleucine	C_6_H_13_NO_2_	amino acid	136.5	1.134	131.09453	Antimicrobial	[46]
l-Phenylalanine	C_9_H_11_NO_2_	amino acid	164.2	1.375	165.07883	Antioxidant, anticancer	[47]
Guvacoline	C_7_H_11_NO_2_	pyridine alkaloid	145.2	1.539	141.07878	-	-
Maltol	C_6_H_6_O_3_	sugar	130.4	2.278	126.03161	Antioxidant	[48]
8-Hydroxyquinoline	C_9_H_7_NO	alkaloid	153.2	2.965	145.05255	Antimicrobial	[49]
4-Hydroxyphenylacetic acid	C_8_H_8_O_3_	benzenoid	156.3	3.314	152.04714	Antimicrobial, antioxidant	[50]
3-Methylcrotonylglycine	C_7_H_11_NO_3_	amino acid	152.6	3.325	157.0737	-	-
Coumarin	C9H6O2	phenol	148.6	3.784	146.0365	Antibacterial, antibiofilm	[51]
Kynurenic acid	C_10_H_7_NO_3_	quinoline carboxylic acid	181.7	3.823	189.04239	Anticancer	[52]
Chlorogenic acid	C_16_H_18_O_9_	phenol	360.5	4.646	354.09435	Anticancer, antioxidant, antibacterial, antiviral, antiobesity and anti-inflammatory	[53]
7-Hydroxycoumarine	C_9_H_6_O_3_	phenol	169.4	4.651	162.0314	Antioxidant, antinociceptive, anti-inflammatory, antitumor, immunomodulator	[54,55]
Caffeic acid	C_9_H_8_O_4_	phenol	186.2	4.692	180.04181	Antioxidant, anti-inflammatory, anticancer and antineoplastic	[56]
Pulegone	C_10_H_16_O	terpenoid	158.9	4.94	152.11989	Antibacterial	[57]
Citral	C_10_H_16_O	terpenoid	145.8	5.575	152.11989	*-*	-
Scopoletin	C_10_H_8_O_4_	coumarin	196.3	5.969	192.04198	Anticancer, antimicrobial	[58]
Isoferulic acid	C_10_H_10_O_4_	phenol	198.6	6.367	194.05762	Antioxidant, antibacterial	[59]
Methyl cinnamate	C_10_H_10_O_2_	cinnamic acid ester	164.5	6.458	162.06775	Antimicrobial, anticancer	[60]
4-Hydroxycoumarin	C_9_H_6_O_3_	benzopyrone	158.8	6.568	162.0314	Antibacterial, antioxidant, antitumor, anti-inflammatory	[56]
Norharman	C_11_H_8_N_2_	alkaloid	164.7	6.725	168.06847	Antimicrobial	[61]
4-Methoxycinnamic acid	C_10_H_10_O_3_	phenol	186.2	7.754	178.06275	Antibacterial	[62]
Rutin	C_27_H_30_O_16_	flavonoid	615.4	8.291	610.15239	Antimicrobial, antioxidant, anticancer, anti-inflammatory, antidiabetic and antiallergic	[63]
Vitexin	C_21_H_20_O_10_	flavonoid	436.8	8.319	432.10497	Antimicrobial, antioxidant, antitumor	[63]
Quercetin	C_15_H_10_O_7_	flavonoid	308.6	8.414	302.04192	Antioxidant, anti-inflammatory, antimicrobial, anticancer, antidiabetic	[64]
Quercetin-3β-d-glucoside	C_21_H_20_O_12_	flavonoid	260.3	8.438	464.09465	Antioxidant	[65]
α-Pinene-2-oxide	C_10_H_16_O	terpenoid	148.9	8.494	152.11989	-	-
Sedanolide	C_12_H_18_O_2_	isobenzofuran	199.5	8.578	194.13039	Anticancer, antioxidant	[66]
Kaempferol	C_15_H_10_O_6_	flavonoid	294.6	8.662	286.04726	Antimicrobial, antioxidant, anticancer, anti-inflammatory, antidiabetic, antiallergic, antiosteoporotic, anxiolytic, analgesic, and antiallergic	[67]
Kuromanin	C_21_H_20_O_11_	chloridis	458.1	8.976	448.09996	Anticancer	[68]
Cycloheximide	C_15_H_23_NO_4_		274.5	10.281	281.16216	Antibacterial	[69]
(-)-Caryophyllene oxide	C_15_H_24_O	epoxide	228.4	10.861	220.18227	Antioxidant, anticancer	[70]
Glycitin	C_22_H_22_O_10_	isoflavone	442.2	11.088	446.12065	Antioxidant, antibacterial	[71]
Apigenin	C_15_H_10_O_5_	flavone	272.4	11.177	270.05235	Anticancer, antibacterial, antioxidant	[72]
Luteolin	C_15_H_10_O_6_	flavonoid	278.6	11.735	286.04723	Antioxidant, anticancer and anti-inflammatory	[73]
Formononetin	C_16_H_12_O_4_	phenol	265.3	12.157	268.07329	Antioxidant, anticancer	[74]
Rhamnetin	C16H12O7	flavonoid	311.2	12.452	316.05766	Antioxidant, anti-inflammatory, cardioprotective, anticancer	[75]
Glycitein	C_16_H_12_O_5_	flavonoid	288.9	13.477	284.06807	-	-
2-Amino-1,3,4-octadecanetriol	C_18_H_39_NO_3_	phenol	311.5	13.499	317.29231	-	-
Diosmetin	C_16_H_12_O_6_	flavonoid	294.3	13.534	300.06279	Antioxidant	[76]
Genistein	C_15_H_10_O_5_	isoflavone	275.3	13.542	270.05214	Antimicrobial, antioxidant, anticancer	[77]
Valine	C_5_H_11_NO_2_	amino acid	118.4	13.901	117.07901	-	-
2-Arachidonoyl glycerol	C_23_H_38_O_4_	fatty acid derivative	371.0	16.012	378.27635	Antimicrobial	[78]
4-Coumaric acid	C_9_H_8_O_3_	phenol	168.2	18.265	164.04718	Antibacterial, antioxidant, antitumor, antimutagenic	[79,80]
Arachidonic acid	C_20_H_32_O_2_	polyunsaturated fatty acid	301.8	18.601	304.2397	-	-
Hexadecanamide	C_16_H_33_NO	fatty acid amide	250.4	19.849	255.25575	-	-
Oleamide	C_18_H_35_NO	fatty acid	286.1	20.177	281.27135	Antimicrobial, anticancer	[81]
Hordenine	C_10_H_15_NO	alkaloid	167.2	23.071	169.23958	-	-
Urocanic acid	C_6_H_6_N_2_O_2_	monocarboxylic acid	131.2	24.845	138.04267	Antioxidant, anticancer	[82]

**Table 3:** Absorption, distribution, metabolism, excretion and toxicity (ADMET) properties of some top identified phytocompounds.

Entry	22	33	37	53
**Druglikeness**				
Lipinski	Yes	Yes	Yes	Yes
Bioavailability Score	0.55	0.55	0.55	0.55
**Absorption**				
Water solubility	−2.504	−3.068	−4.321	−1.219
Caco2 permeability	1.184	1.616	1.414	1.587
Intestinal absorption (human)	95.277	96.423	95.669	93.396
Skin Permeability	−2.944	−2.237	−3.061	−2.506
P-glycoprotein substrate	No	No	No	No
P-glycoprotein I inhibitor	No	No	No	No
P-glycoprotein II inhibitor	No	No	No	No
**Distribution**				
VDss (human)	0.034	0.31	0.564	0.887
BBB permeability	−0.299	0.591	0.647	-0.083
CNS permeability	-2.32	−2.511	−2.521	−1.75
**Metabolism**				
CYP2D6 substrate	No	No	No	Yes
CYP3A4 substrate	No	No	No	No
CYP1A2 inhibitior	Yes	No	Yes	No
CYP2C19 inhibitior	No	No	Yes	No
CYP2C9 inhibitior	No	No	Yes	No
CYP2D6 inhibitior	No	No	No	No
CYP3A4 inhibitior	No	No	No	No
**Excretion**				
Total Clearance	0.73	1.356	0.905	0.907
Renal OCT2 substrate	No	No	No	Yes
**Toxicity (Compounds number)**				
AMES toxicity	3 (10, 26, 27)			
Hepatotoxicity	3 (9, 25, 50)			
hERG I inhibitors	No			
Skin Sensitisation	14 (2, 4, 5, 10, 20, 24, 32, 33, 37, 44, 48, 50, 51, 52)			

22: Scopoletin, 33: Sedanolide, 37: (-)-Caryophyllene oxide, 53: hordenine. VDss: volume of distribution at steady state; BBB: brain blood barrier; CNS: central nervous center; CYP: cytochrome P; OCT: organic cation transporter.

## Data Availability

All data generated or analyzed during this study are included in this article.

## Sample Availability

Sample of the extract is available from the authors.
